# Climate Change—The Rise of Climate-Resilient Crops

**DOI:** 10.3390/plants13040490

**Published:** 2024-02-08

**Authors:** Przemysław Kopeć

**Affiliations:** The Franciszek Górski Institute of Plant Physiology, Polish Academy of Sciences, Niezapominajek 21, 30-239 Kraków, Poland; p.kopec@ifr-pan.edu.pl

**Keywords:** multifactorial stress, GE interaction, crop domestication

## Abstract

Climate change disrupts food production in many regions of the world. The accompanying extreme weather events, such as droughts, floods, heat waves, and cold snaps, pose threats to crops. The concentration of carbon dioxide also increases in the atmosphere. The United Nations is implementing the climate-smart agriculture initiative to ensure food security. An element of this project involves the breeding of climate-resilient crops or plant cultivars with enhanced resistance to unfavorable environmental conditions. Modern agriculture, which is currently homogeneous, needs to diversify the species and cultivars of cultivated plants. Plant breeding programs should extensively incorporate new molecular technologies, supported by the development of field phenotyping techniques. Breeders should closely cooperate with scientists from various fields of science.

## 1. Introduction

Climate change is one of the challenges facing modern agriculture, compounded by the global population growth and soil quality deterioration. These problems are interrelated, making it difficult to counteract them. The grand challenge of future agriculture is to introduce a sustainable “new Green Revolution”. The Green Revolution in the 20th century provided greater food security in developing countries by introducing improved cultivars with a high yield potential and agrotechnological recommendations, entailing intensive fertilization with mineral fertilizers, pesticide use, and the development of irrigation systems, among other practices. However, we are currently grappling with the long-term negative effects of this progress. The expansion of the high-input agriculture model and the Industrial Revolution led to environmental chemization, water pollution, the loss of biodiversity, and soil degradation [1,2]. The modern agriculture development strategy must include a better match of crops with their environment, considering climate change, a reduced use of pesticides and fertilizers, and a focus on the quality of yield, not only its quantity, to ensure nutritional stability [3].

**Figure 1:**
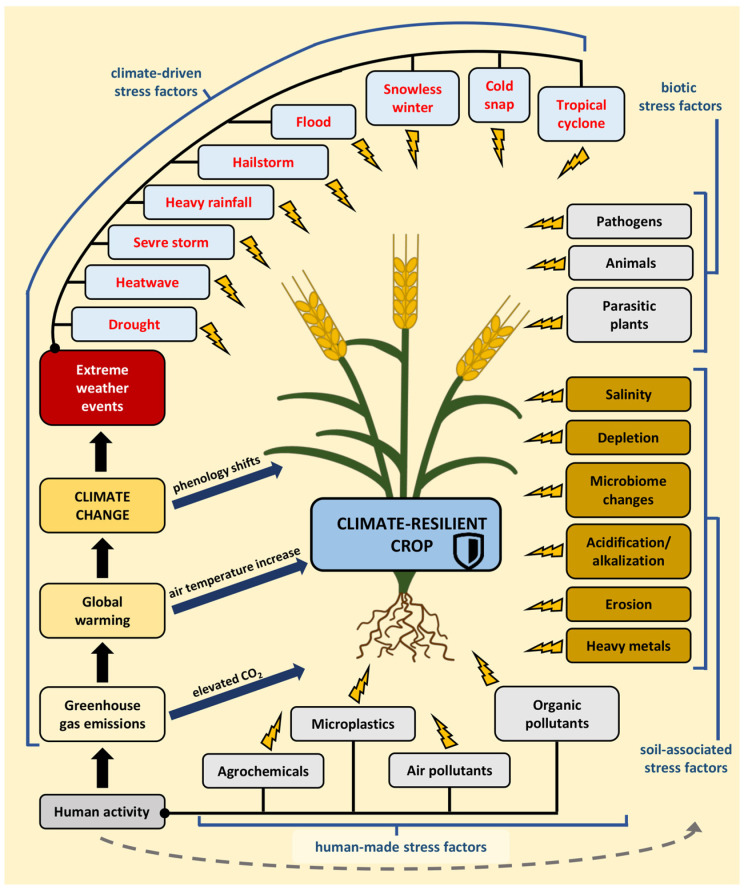
A climate-resilient crop is resistant to multiple stress factors, which can be abiotic, including both human-made and climate-driven factors, as well as soil-associated or biotic stress factors. Human activity generates greenhouse gas emissions, including carbon dioxide, that accelerate global warming, leading to climate change. This, in turn, could impact the frequency of extreme weather events and shifts in plant phenology. Human activity also influences the soil and exacerbates soil-associated stresses.

Climate change is a modification in the statistical properties of the climate system that persists for an extended period. These statistical properties encompass the mean and variability. Changes in a climate may stem from natural internal processes or external forces, such as anthropogenic influences on the atmosphere’s composition or land use [4]. Ongoing climate change is primarily driven by human-induced global warming (Figure 1). Since the Industrial Revolution, increasing human activity has generated greenhouse gas (GHG) emissions and aerosols altering the Earth’s surface reflectivity through changes in land cover. Greenhouse gases in the atmosphere, methane, nitrous oxide and especially carbon dioxide (CO_2_), trap heat emitted from the Earth’s surface after absorbing sunlight, leading to an increase in temperature of the surface of the planet, lower atmosphere, and oceans. GHGs are primarily produced during the combustion of fossil fuels. The last four decades have been warmer than the previous decades since 1850. By comparing the periods 1850–1900 and 2010–2019, the global increase in surface temperature can be observed to be approximately 1.07 °C [5]. However, climate change is not solely associated with a rise in ambient temperature, but also with an increase in precipitation variability and the frequency of extreme weather events such as droughts, floods, heatwaves, and cold snaps [6,7].

Climate change has far-reaching consequences for agriculture. It alters the distribution of crops and the suitability of growing areas and leads to shifts in plant phenology [8,9]. Higher temperatures and increased surface CO_2_ levels can enhance crop yields in certain locations, contingent upon meeting other requirements such as nutrient levels, soil moisture, and water availability. Despite the potential of elevated CO_2_ to stimulate plant growth, it can also diminish yield quality [10,11,12,13]. In regions situated at the highest latitudes of the northern hemisphere, where agriculture is presently limited by cold temperatures, climate warming can improve agriculture productivity and efficiency [2]. This also creates the possibility of expanded farming in Arctic areas [9]. However, in many parts of the world, the increased frequency and severity of extreme weather events have reduced the productivity of major food and feed crops, posing a significant threat to regional and global food security [6]. Furthermore, warmer temperatures and elevatedCO_2_ levels promote the development of weeds, pests, and pathogens [10,14,15].

The United Nations has launched an initiative to ensure food stability and security through the implementation of climate-smart agriculture (CSA). This approach guides actions necessary to transform and reorient agricultural systems to sustainably increase agricultural productivity and incomes, build resilience and adaptation to climate change, and reduce and/or remove greenhouse gas emissions where possible [16]. One pathway to achieve these goals is through climate-resilient crops. These crops or plant cultivars exhibit enhanced resistance to adverse environmental conditions, with the intention of maintaining or increasing crop yields under stress conditions [17] (Figure 1).

This review introduces climate-resilient crops—an idea gaining importance in light of accelerating climate change and its negative effects on humans, especially in the area of food production. The review emphasizes the significance of this idea and outlines the challenges it faces. The implementation of climate-resilient crops in general agriculture poses a major challenge for breeders.

## 2. Crops under Multiple Stresses

Plants can remain under the influence of multiple stress factors, which may be abiotic, including both human-made, climate-driven, and soil-associated or biotic [18] (Figure 1). Often, plants are subjected to several stress factors simultaneously, and these factors can interact with each other. The nature of this interaction can be synergistic, antagonistic, or additive. Synergy occurs when the combined effect of multiple stressors exceeds the sum of the effects of each individual stressor. Conversely, antagonistic interactions happen when the combined effect of the stressors is less than the sum of the effects of each individual stressor. Additive interaction implies that the combined effect of many stressors is equal to the effect of their sum. The type of stress interaction depends not only on the factors creating them but also on the plant they affect. A stress combination can synergistically influence one species but antagonistically affect another [19].

Stresses cause a breakdown in plant homeostasis, affecting metabolic and physiological processes. Plants have the ability to establish a new state of homeostasis, rapidly adapting to environmental changes during their lifetime. This adaptive process is referred to as acclimation. Over an extended period, plants can also adapt to a new environmental pattern due to hereditary changes in the population resulting from alterations in the genome; this is known as adaptation [20].

**Figure 2:**
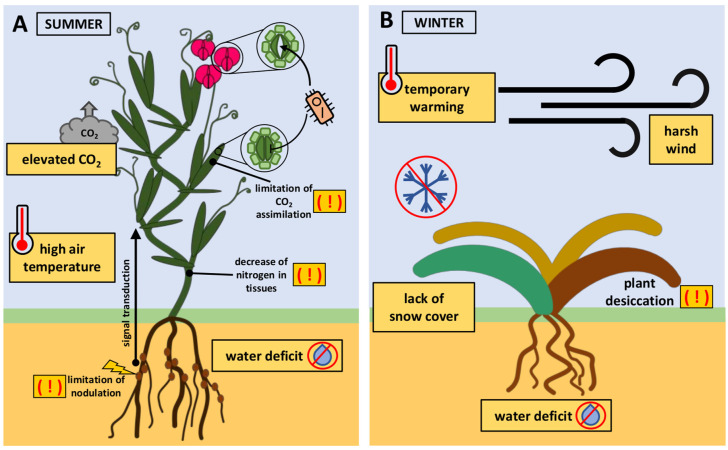
Examples of the implications of the simultaneous impact of many stress factors on crops. (**A**) Soil water deficit triggers signals from roots to shoots. Consequently, the stomata in the leaves close. However, high air temperatures stimulate stomatal opening in flowers. Stomatal closure limits pathogen transmission, while open stomata present a potential route of infection for weakened plants due to unfavorable environmental conditions. In plants with C_3_ photosynthesis, elevated concentrations of CO_2_ (e[CO_2_]) in the atmosphere do not stimulate carbon assimilation. Drought and e[CO_2_] disturb legume nodulation and lead to a decrease in nitrogen in tissues. (**B**) The lack of rainfall in autumn may result in water deficiency in the soil during winter. Winter crops may experience temporary warming in the middle of winter, causing the plants to reduce their winter hardiness due to the deacclimation process. The accompanying lack of snow cover and harsh winds result in the desiccation of plants.

Much research work is focused on plant responses to a single stressor or to simple stress combinations of two or, at most, three different stressors occurring simultaneously. However, in nature, plants are routinely subjected to multiple stresses (Figure 1). The simultaneous impact of three or more stresses on a plant can be defined as a multifactorial stress compilation [18]. The most commonly studied simple combination of stresses is soil drought and high air temperature (Figure 2A). During water deficits, roots transmit signals to the shoot, triggering stomatal closure. Stomatal closure prevents water loss from the tissues. On the contrary, warm temperatures stimulate stomatal opening, allowing plants to cool themselves through transpiration. The simultaneous occurrence of both stresses often results in stomatal closure in the leaves but not in the flowers, where they remain open. This prevents tissue overheating and protects the reproductive process. This acclimation strategy is termed ‘differential transpiration’ [21]. The stomatal closure of leaves has an advantage because it limits pathogen transmission to the leaf, which is particularly exposed to plants under unfavorable conditions [15]. The combination of drought and heat stress is now accompanied by elevated concentrations of CO_2_ (e[CO_2_]) in the atmosphere. In crops with C_3_ photosynthesis, e[CO_2_] causes increased photosynthetic carbon assimilation and reduced stomatal conductance. Stomatal closure results in the lack of stimulation of the intercellular CO_2_ concentration of leaves necessary for improved carbon assimilation at e[CO_2_]. The combination of soil water deficit with e[CO_2_] disturbs legume nodulation and leads to a decrease in nitrogen in tissues [22]. The rising atmospheric CO_2_ concentration generally does not directly stimulate C_4_ crop photosynthesis in optimal growth conditions. However, e[CO_2_] can improve the crop yield in areas where water is limited [23] and also reduce the negative impact of heatwaves [24]. Low access to nitrogen in the soil with e[CO_2_] may enhance increased extraradical mycorrhizal hyphal density. Mycorrhizal fungi live in symbiosis with plant roots, obtaining carbon from host plants and providing nutrients and other benefits, such as protection against pathogens [25].

Another example of the problem of multifactorial stress occurs in winter crops cultivated under temperate climate conditions (Figure 2B). The seeds of these plants are sown in the autumn and germinate before winter arrives. Plants acquire increased tolerance to freezing through prior exposure to low, non-freezing temperatures. This adaptive process is termed cold acclimation [26]. Global warming indicates a lower risk of exposure of winter crops to extremely low temperatures. However, the risk of winter damage to crops may not decrease due to complex interactions among environmental factors. In winter, there may be extreme weather events, no snowfall, or temporary warming leading to snowmelt. This may increase the risk of freezing injury in crops that are not covered with snow. Furthermore, temporary warming during winter can cause deacclimation, eventually reducing winter hardiness [27]. Often, the situation is further complicated by winter drought of the soil [28], which, combined with harsh winds, causes winter desiccation [29].

The occurrence of two or more different stressors may necessitate various and sometimes opposite response mechanisms. In such conditions, the stress response in plant can involve choosing one pathway as being dominant over the others or using a combination of responses to create a completely new reaction strategy. The plant’s reaction strategy depends on the sequence in which stresses occur, which stress appeared first and which occurred during ongoing stress [7]. Studies on the effect of a combination of six abiotic stress conditions on Arabidopsis (*Arabidopsis thaliana*) seedlings revealed that while each of the different stresses (heat, salt, high light, cadmium, acidity, herbicide paraquat), applied individually, had a negligible effect on plants, the interaction of stresses and the sum of their effects made them considerably harmful [30]. Low levels of stress factors, considered individually, might be overlooked when predicting their impact on crop yields. However, their interaction can lead to a substantial decrease in agricultural productivity [7].

The various stress factors that simultaneously impact a plant may be sensed by the same or different parts of plants; the entire plant senses heat stress, the roots sense heavy metals in the soil, and the leaves sense powdery mildew. Plants receive external stimuli in different ways, engaging more or less specific receptors and signal transduction networks. Each stress triggers its own specific systemic signaling and acclimation responses including physiological and metabolic reactions. Plants can integrate systemic signals, and their response will depend on how plants sense the factors causing these signals [7,31].

The high yield and quality in the vast majority of cultivated plants depend on the quality of the soil. The effects of stress factors associated with climate change and human activity were studied in soils using different combinations of drought, low nitrogen, temperature, microplastics, glyphosate, antibiotics, fungicides, copper, salinity, and insecticides. An increase in the number of simultaneous factors constituting a multifactorial stress combination was found to intensify negative effects on soil properties and its microbiome [32,33]. Many factors that negatively affect plants also negatively impact the soil [34,35]. Thus, the deterioration of soil properties is an additional factor that stresses crops.

## 3. Challenges for Modern Plant Breeding

### 3.1. Genotype × Environment Interaction

Breeders have the responsibility of producing cultivars that meet the local needs of farmers and also exhibit a wide enough adaptation to justify the investment and scale of their selection programs. To address this challenge, they must select new distinctive cultivars that are adapted to a much wider range of environments than those that have been tested [3]. The presence of genotype × environment (GE) interactions remains a significant impediment for plant breeders, geneticists, and agronomists conducting multisite trials to evaluate crop performance. GE interactions complicate and can impede progress in selecting a genotype for a target trait, as various genotypes respond differently and with varying intensities under different environmental conditions [36]. Breeders typically rank tested genotypes at different sites based on the averages of the traits under consideration. Ideally, the rank orders of the genotypes should remain constant across various locations. However, GE interactions disturb this state and cause a change in rank among genotypes across locations, making it challenging to identify significantly superior genotypes [37]. There are two potential strategies to reduce the GE interaction in plant breeding. The first involves the stratification of environments, where breeding regions are subdivided into subregions with similar conditions. Breeding for specific adaptation to subregions, where genotype responses are relatively uniform, may minimize genotype x location (GL) interaction, which is part of the GE interaction. The second strategy involves selecting stable genotypes that interact less with the environments in which they are grown [38,39].

GE interactions can be either qualitative (i.e., crossover type) or only quantitative (i.e., non-crossover type). Crossover interactions represent differences in genotype rankings and are of particular concern for breeders as they have the potential to jeopardize the selection of superior genotypes and may provide fewer genetic benefits as more crosses occur. Non-crossover interactions are associated with changes in relative yields but do not lead to significant rank changes between genotypes within environments [40]. Understanding the causes of the GE interaction can be used to establish breeding purposes, identify ideal test sites, and formulate recommendations for cultivation regionalization [41]. Defining patterns of relationships between environmental variables and phenotypes through genetic analysis will enhance our understanding of the GE interaction [42]. A combination of genotypic and phenotypic profiling of genetic resource collections is achieved in pre-breeding steps and during research [3].

One of the main reasons for the slow progress in breeding climate-resilient crops is that most of the important characteristics of crops are polygenic traits [43,44]. Additionally, most quantitative trait loci (QTLs) or genes have been tested under controlled growth conditions, which do not reflect real field conditions [45]. For exploring multiple traits in plants and genetic sources of phenotypic variation, QTL mapping and genome-wide association studies (GWASs) are used. QTL mapping is a statistical method that links traits with specific regions of chromosomes, but it is limited in terms of allele diversity and genomic resolution. However, GWAS provides higher resolution, often to the gene level. This technology evaluates a single nucleotide polymorphism (SNP) for association with a phenotype. The disadvantages of GWAS include its low power to map multiple functional alleles within one gene and its inefficiency in identifying rare alleles within a population [46,47,48].

The progress in breeding has been hampered by the lack of high-performance and non-destructive tools for plant phenotyping in field conditions. In recent years, field phenomics has emerged in response to the demand for high-throughput phenotyping (HTP). It relies on imaging and sensor technologies that allow rapid and cost-effective measurements with less labor. However, in many cases, these devices require high initial investments and are not yet widespread. High-throughput phenotyping platforms enable the consideration of morphological and physiological traits such as green area indexes (GAIs), chlorophyll content, nitrogen content, plant density at emergence, ear density, grain number and size, fraction of absorber photosynthetically active radiation (FAPAR), staygreen/senescence, crop dynamics monitoring, phenology, canopy coverage, plant health, canopy height, canopy temperature, leaf rolling, leaf angle, leaf wilting, lodging, chlorophyll fluorescence, photosynthetic status, biomass, water content, grain quality, water use efficiency, canopy structure, weed infestation, light use efficiency, nitrogen use efficiency, root development and yield [45,49,50]. Progress in HTP is attributed to the rapid development of sensor technologies, including red–green–blue (RGB), multispectral, hyperspectral, and thermal cameras; photosynthesis and fluorescence sensors; stereo cameras; and LiDAR devices [49,51,52,53,54]. For phenotyping, ground- and aerial-based platforms can be used, with sensors installed on stationary or mobile platforms, including handheld devices. Aerial-based technologies encompass satellite, manned, and unmanned aerial vehicles [3,45].

### 3.2. Crop Domestication

The worldwide crop structure is homogeneous and uniform. Global demand for food is primarily satisfied by rice, wheat, and corn, contributing to 60% of the calories consumed by humans [55,56]. However, this situation poses a threat to crops due to the widespreading diseases and pests or potential yield loss caused by changing environmental conditions.

Cultivated crops were domesticated from wild plants. During the domestication process, attractive human allelic variants were selected, while some remaining variants were not fixed and do not exist in the current cultivars. Domesticated or semi-domesticated plants were widespread, harboring allelic variants suitable for local environments. Traits lost over time can play a role in adapting cultivated species to specific climatic niches [46,57,58,59].

In the development of climate-resilient crops, attention has been given to wild, semi-domesticated, and orphan plants. Semi-domesticated crops are plants that were cultivated and then reverted to a wild status and neglected cultivar plants that were initially adapted for agriculture but were subsequently abandoned in their further development. Orphan plants, on the other hand, are wild plants, usually known only locally, that have not undergone intensive artificial selection [46,60]. Semi-domesticated and orphan plants typically exhibit low productivity and cannot be grown on a large agricultural scale. However, breeding these plants can provide crops adapted to local conditions, serving as a valuable food source for local communities. Wild, semi-domesticated and orphan plants, due to their tolerance of various unfavorable environmental conditions, can serve as a source of germplasm in the breeding of high-production stress-tolerant crops for large-scale agriculture [46]. Currently, efforts are being made to reintroduce the beneficial traits lost during artificial selection into staple crops by redomesticating crop wild relatives [57]. In conventional breeding, the process of crossing crop plants with their wild relatives is problematic in many cases. Their biology may not be in sync with each other, and there is also the possibility that stress-adaptive traits of wild plants may negatively impact favorable agronomic traits [3,61,62].

One possibility for building sustainable agriculture that is being explored is the development of perennial grain cropping systems. Tolerance to unfavorable environmental conditions, the efficient use of nutrients and water, and soil conservation are some of the potential benefits of replacing annual food crops with perennial alternatives, particularly in erodible and marginal sites. The process of changing the cultivation system begins by crossing annual crops with their perennial wild relatives. Currently, the agronomic traits of such plants and their productivity are not yet satisfactory [63,64], but they have potential to become a part of diversified agriculture.

Progress in the domestication of current plants has been facilitated by the identification of many domestication genes. This success is attributed to the development of new technologies. Advanced genomic tools can now be employed to support artificial selections [58,65,66].

A strategy for improving crops, suitable for the environment and the needs of agricultural production, requires the identification of specific beneficial alleles responsible for desirable variations in traits and the removal of deleterious variants. The effective creation of climate-resilient crops with various alleles for target genes has become technically feasible. Achieving this goal requires the use of state-of-the-art technologies, such as advanced genome sequencing pipelines, big data deep learning, precise genome editing tools, synthetic biology methods, and the previously mentioned high-throughput phenotyping [46,67].

Allelic variation underlying important agronomic traits and its incorporation into breeding programs are explored and exploited with genomics-assisted breeding (GAB) [68,69]. Genetic studies on crop domestication and improvement are based on reference genome sequences of relevant plant species. The breakthrough next-generation sequencing (NGS) technology, which relies on the massive sequencing of short sequences, is being replaced by third-generation sequencing (TGS) that generates long-sequence reads. The development of sequencing technology is accompanied by progress in the design of algorithms and tools for processing the large data received. Although current reference genomes have gaps, such as at telomere sites and highly polymorphic regions, efforts are already being made to complete the information on this subject [58,70,71]. Along with the progress of sequencing technology and bioinformatics, crop population genomics is transforming from a single reference resequencing approach into a pangenome approach. The pangenome refers to the totality of genome sequences existing in one studied species. Additionally, the superpangenomic approach is being developed, referring not only to the species but to the entire genus. By constructing more genome sequences representative of a species or genus, it will be possible to capture a holistic picture of genetic diversity spanning the entire gene pool of crops [58,68,72,73]. Long-read sequencing, in combination with improved haplotype resolution for complete genome assemblies, helps to create platinum standard reference genome sequences (PSRefSeq). PSRefSeq can be used as a template to map resequencing data to detect structural variations (SVs), including insertion/deletion (InDEL), copy number variation (CNV), and presence–absence variation facilitate the cataloguing of structural variations (SVs), including insertion/deletion (InDEL), copy number variation (CNV), and presence–absence variation [68,74,75].

Current breeding demands quick and precise actions. Genomic breeding (GB) tools come in handy, including marker-assisted selection (MAS), marker-assisted backcrossing (MABC), marker-assisted recurrent selection (MARS), haplotype-based breeding (HBB), and the promotion/removal of alleles through genome editing (PAGE/RAGE) [68,69,76,77,78].

## 4. Climate-Resilient Crops

This chapter is dedicated exclusively to crop species that fit the idea of building climate resilience. These plants contribute to the diversification of global crops. The staple crop cultivars will not be listed. It is important to note that the primary focus of plant breeding activities remains to be the improvement staple crops. The climate-resilient crops discussed below have low cultivation requirements, enabling their use on marginal farmland without the need for high-input fertilization. They are usually tolerant to periods of soil water deficit.

Pearl millet (*Pennisetum glaucum* L.; *Poaceae* family) is a widely embraced plant in Sub-Saharan Africa. Its notable advantage lies in its tolerance to prolonged periods of water deficits in the soil and high temperatures. It grows in areas with increased salinity, even enduring drought and extreme heat during the reproductive phase [79].

It is noteworthy to mention sorghum (*Sorghum bicolor* L.; *Poaceae* family) here, despite being one of the most important carbohydrate-rich crops globally, following wheat, corn, barley, and rice. Sorghum serves as a staple food for people in Asia and Africa and is also a major feed crop in many countries worldwide. Its adaptability to in semi-arid regions and areas with irregular rainfall makes it a preferred choice, owing to its tolerance for soil drought and high air temperature. Additionally, it can be cultivated in low-input marginal farmland [80].

In Central and Eastern Europe, rye (*Secale cereale* L.; *Poaceae* family) is widely cultivated. This cereal exhibits high tolerance to location, forecrop, and fertilization and displays resistance to various diseases and adverse environmental conditions [81]. Recently, there has been a growing interest in primitive rye, *Secale cereale* var. *multicaule*, particularly for grain production in mountainous regions due to its frost resistance and tolerance to poor soil conditions [82,83]. Primitive rye is a biennial plant that, when sown in spring, allows for biomass harvesting for silage in the same year and seed harvesting the following year. Alternatively, when sown in autumn, it is cultivated like winter rye [83]. The plant’s excellent tillering ability effectively suppresses weeds. It can also be used to regenerate agricultural soils [82].

Amaranth (*Amaranthus* spp. L.; *Amaranthaceae* family), projected as a future crop, is a pseudocereal known since ancient times in Central and South America. Three cultivated species include *A. cruentus* L., *A. caudatus* L., and *A. hypochondriacus* L. [84]. Amaranth stands out for its high genetic diversity and extensive phenotypic plasticity, making it well-adapted for cultivation in nutrient-poor soils. It exhibits tolerance to soil water deficit, high soil salinity, and high air temperatures. Furthermore, the cultivation of amaranth is not threatened by pests and diseases [85,86,87,88,89].

Another pseudocereal with substantial resilience to climate change, originating from the same region as amaranth, is quinoa (*Chenopodium quinoa* Willd.; *Amaranthaceae* family). Although its cultivation is primarily concentrated in South America, quinoa can grow in rocky and low-input fields. It demonstrates tolerance to cold temperatures, soil salinity, and water deficit, featuring deep rooting systems and high water-use efficiency [90].

Receiving increasing attention is an old European crop, camelina (*Camelina sativa* L.; *Brassicaceae* family), which is valued for its flexibility to adapt to diverse cultivation conditions. Camelina grows in various climatic and soil conditions, excluding heavy clay and organic soil. It yields successfully even when other crops fail. It serves as an oilseed crop in food, feed, and biomedical industries and the production of low-emission biodiesel fuel. Camelina is adapted to cool temperate semi-arid climates, presenting itself as a low-input crop with tolerance to soil drought and low rainfall and a short vegetation period [91,92,93].

## 5. Conclusions

Climate change poses a threat to food security in many regions of the world. Therefore, it is crucial today to improve crops that contribute to the development of climate-smart agriculture initiatives. The presence of extreme weather events accompanying climate change directly leads to reduced crop yields. Climate-resilient crops and cultivars offer a solution for how farmers can cope with climate change. These crops yield stably in new environmental conditions, preventing productivity decline and crop failure. The task of “up-grading” crops has become a grand challenge for breeders, extending beyond mere economic interests to encompass national and global security.

Plant breeding requires thoughtful scientific support. In recent years, technological advancements have rapidly boosted fundamental scientific discoveries. The application of scientists’ achievements in breeding programs can be challenging. Even research conducted with breeders in mind often occurs under controlled conditions, with results not adequately tested in the field. The response of plants to stress treatment in simulated environmental conditions, especially when examined individually, differs from real field conditions, where crops face simultaneous exposure to abiotic and biotic factors.

The development of climate-resilience crops necessitates predicting and identifying future agricultural problems from both local and global perspectives. Understanding the impact of multifactorial stress on cultivated plants, their wild relatives, and semi-domesticated plants is crucial. To improve food security, global plant cultivation must diversify through the dissemination of new crops or the generation of improved cultivars of staple crops.

Plant breeding should extensively leverage new molecular technologies for long-term and multi-site field trials. Further development of high-performance and nondestructive field phenotyping techniques is necessary to facilitate rapid progress. Close collaboration between breeders and scientists specializing in genetics, physiology, proteomics, metabolomics, agronomy, and meteorology, as well as with engineers and big data specialists, is essential.

## Data Availability

Not applicable.
